# Amyloid Beta Detection by Faradaic Electrochemical Impedance Spectroscopy Using Interdigitated Microelectrodes

**DOI:** 10.3390/s18020426

**Published:** 2018-02-01

**Authors:** Jin Soo Park, Hye Jin Kim, Ji-Hoon Lee, Jung Ho Park, Jinsik Kim, Kyo Seon Hwang, Byung Chul Lee

**Affiliations:** 1 Center for BioMicrosystems, Korea Institute of Science and Technology (KIST), Seoul 02792, Korea; akadk2@kist.re.kr (J.S.P.); ghooneee@gmail.com (J.-H.L.); 2 Department of Clinical Pharmacology and Therapeutics, College of Medicine, Kyung Hee University, Seoul 02447, Korea; hyejinkim.mail@gmail.com; 3 Department of Electrical Engineering, Korea University, Seoul 02841, Korea; jhpark@korea.ac.kr; 4 Department of Medical Biotechnology, College of Life Science and Biotechnology, Dongguk University, Seoul 10326, Korea; lookup2@dongguk.edu

**Keywords:** amyloid beta, redox reagent, [Fe(CN)_6_]^3−/4−^, faradaic electrochemical impedance spectroscopy, biosensor, high sensitivity

## Abstract

Faradaic electrochemical impedance spectroscopy (f-EIS) in the presence of redox reagent, e.g., [Fe(CN)_6_]^3−/4−^, is widely used in biosensors owing to its high sensitivity. However, in sensors detecting amyloid beta (Aβ), the redox reagent can cause the aggregation of Aβ, which is a disturbance factor in accurate detection. Here, we propose an interdigitated microelectrode (IME) based f-EIS technique that can alleviate the aggregation of Aβ and achieve high sensitivity by buffer control. The proposed method was verified by analyzing three different EIS-based sensors: non-faradaic EIS (nf-EIS), f-EIS, and the proposed f-EIS with buffer control. We analyzed the equivalent circuits of nf-EIS and f-EIS sensors. The dominant factors of sensitivity were analyzed, and the impedance change rates via Aβ reaction was compared. We measured the sensitivity of the IME sensors based on nf-EIS, f-EIS, and the proposed f-EIS. The results demonstrate that the proposed EIS-based IME sensor can detect Aβ with a sensitivity of 7.40-fold and 10.93-fold higher than the nf-EIS and the f-EIS sensors, respectively.

## 1. Introduction

Electrochemical impedance spectroscopy (EIS)-based biosensors are widely used in food, medical, and environmental analysis since they can be easily miniaturized using microelectromechanical systems (MEMS) technology and directly convert biomolecular reactions to relevant electrical information [1]. EIS-based sensors typically utilize an electrochemical cell that consists of two electrodes (working and counter electrodes) filled with an electrolyte. The electrodes (or insulator) are functionalized with particular bio-receptors for target-specific detection of biomolecules. The binding of target biomolecules (i.e., bio-recognition events) modifies the interfacial properties, causing changes in impedance values. The bio-recognition events such as antigen–antibody complex formation, DNA hybridization, and enzymatic reactions can be quantified by measuring the effective changes of the impedances [2].

EIS-based biosensors can be classified into two types depending on the presence of redox reagents, namely, non-faradaic EIS (nf-EIS)-based and faradaic EIS (f-EIS)-based sensors. The nf-EIS-based sensor uses an electrolyte that does not contain a redox reagent and predominantly depends on the capacitance change at interface between the electrodes and the electrolyte. The f-EIS-based sensor mainly uses the charge transfer process between the electrodes and the redox reagent. By controlling the charge transfer process using a bio-reaction, the f-EIS sensor can achieve a higher sensitivity than nf-EIS [3,4]. However, the incorporation of a redox reagent into the f-EIS sensor makes it difficult to detect specific biomolecules such as amyloid beta (Aβ), which is the main component of amyloid plaques found in the brains of patients who have Alzheimer’s disease [5]. The redox reagent contains metal ions such as aluminum (Al^3+^), iron (Fe^2+/3+^), and zinc (Zn^2+^). These metal ions can promote the Aβ aggregation in buffer solution [6]. In both nf-EIS and f-EIS sensors based on immunobinding of the antigen–antibody complex, the one-to-one reaction of antigen–antibody is critical in accurately measuring the biomarkers. However, the aggregation can disturb the one-to-one reaction of Aβ oligomer with antibodies and disables quantitative detection of Aβ at extremely low concentrations [7]. Therefore, to precisely detect Aβ using f-EIS-based sensors, the aggregation effect must be minimized.

In this study, we propose an aggregation-free f-EIS technique that can achieve the high detection sensitivity of Aβ on an interdigitated microelectrode (IME) sensor. The proposed sensing method alleviates the aggregation of Aβ via a deliberate control of buffer solution that involves a conversion of the buffer containing the [Fe(CN)_6_]^3−/4−^ into a pure buffer without the [Fe(CN)_6_]^3−/4−^ during the reaction of Aβ with its specific antibody, 6E10. The IME sensor was fabricated based on a MEMS process. The sensitivity of the fabricated sensor was verified by measuring the impedance spectrum of the sensor. Before verifying the sensitivity of the proposed f-EIS-based IME sensor, we examined the equivalent circuits of an nf-EIS-based IME sensor and an f-EIS-based IME sensor. The circuit factors affecting the sensitivity of each EIS-based IME sensor were analyzed by measuring the change of each factor from the Aβ reaction. The sensitivities of the three sensors were quantified by analyzing the measured impedance changes by the reaction of various concentration of Aβ. Consequently, we demonstrated that the sensitivity of the proposed f-EIS-based IME sensor was enhanced approximately 7.40-fold and 10.93-fold compared to the f-EIS-based IME sensor and the nf-EIS-based IME sensor, respectively.

## 2. Model Evaluation of Each nf-EIS-based and f-EIS-based IME Sensor

The EIS-based biosensors were classified into two types, non-faradaic and faradaic, depending on the presence or absence of redox reaction. To demonstrate the detection mechanisms, we analyzed the Randles equivalent circuit models of the two methods with the experimentally measured impedance spectra of the two [8].

**Figure 1:**
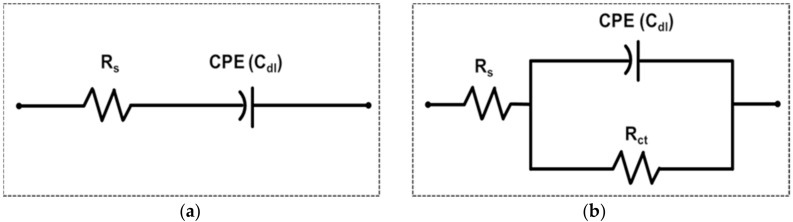
Equivalent circuit model of (**a**) non-faradaic spectroscopy and (**b**) faradaic spectroscopy. CPE (C_dl_) is a constant phase element that represents a double layer capacitance, *R*_s_ is a solution resistance and *R*_ct_ is a charge transfer resistance, respectively.

Figure 1a is the equivalent circuit model of the nf-EIS-based IME sensor. The circuit model is composed of a solution resistance *R*_s_, which represents bulk solution conductivity, and a constant phase element (CPE) connected in series. The CPE represents the electric double layer capacitance at the electrodes–electrolyte interface in EIS and the impedance of CPE can be expressed in Equation (1) [9]. The CPE behavior is generally attributed to the time constant distribution caused by interfacial heterogeneity such as electrode surface roughness [10]. The value of α is an indicator of homogeneity of the interfacial properties and takes a value between 0 and 1. α = 1 implies an ideal capacitor, for which *Q* has a unit of capacitance, farads (F). Otherwise, *Q* has units of Fs^(α−1)^.

(1) $$Z_{\mathrm{CPE}}=\frac{1}{{\left(j2\pi f\right)}^{\alpha }Q}.$$

In the case of nf-EIS, which is performed in the absence of redox reagent, the electrodes are electrically isolated from the faradaic reaction such as charge transfer at the electrodes–electrolyte interface. Thus, the nf-EIS-based detection predominantly depends on the total effective capacitance change (CPE change). The effective capacitance change of the sensor is originated from the bio-recognition events of target molecules, e.g., the antigen–antibody complex. When immuno-binding of antigen to antibody occurs at electrodes or insulator surfaces, the thickness or dielectric properties of electrical double-layer capacitance whose typical value is 10–100 μF/cm^2^ change [11]. However, since the capacitance change by immuno-binding of antigen is typically small when comparing the absolute value of double layer capacitance, the corresponding impedance change is normally less than 10%, and the detection sensitivity of the nf-EIS sensor is inherently low [12,13].

On the other hand, the f-EIS method, which is performed in the presence of redox reagent, can detect the various biomolecules with high sensitivity by measuring the charge transfer process between electrode and redox reagent [3,4]. Figure 1b shows the equivalent circuit of the f-EIS-based IME sensor. The charge transfer resistance, *R*_ct_, results from the charge transfer process between the redox reagent and the electrodes. The charge transfer of the reagent generates a faradaic current depending on the electrode potential and makes a parallel branch with the capacitance, CPE [14].

When antigen–antibody binding occurs, the kinetics of the redox reagents nearby the electrodes are interfered by physical blocking effects of the antigen–antibody complex [15]. This phenomenon suppresses the charge transfer process by the redox reaction of the reagent. The suppressed charge transfer (or equivalently increased *R*_ct_) mainly attributes to the impedance change of the IME sensors; the change of *R*_ct_ is quantitatively measured to determine the concentration of the target molecules. As more antigen reacts with immobilized antibody, the physical blocking effect of the immune complex further increases, and the total effective impedance change increases accordingly. In the f-EIS-based IME sensor, therefore, by controlling the charge transfer process at the electrodes–electrolyte interface by antigen–antibody binding, the substantial impedance change and high detection sensitivity can be achieved.

## 3. The Proposed f-EIS-Based Interdigitated Microelectrode Sensor

**Figure 2:**
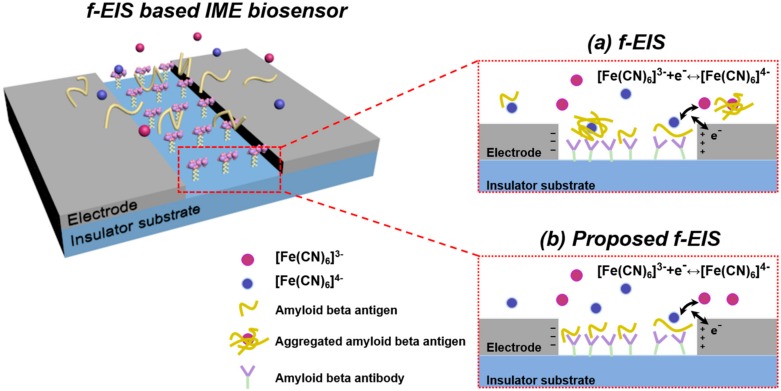
Schematic of Aβ detection in faradaic electrochemical impedance spectroscopy (f-EIS) biosensor (**a**) f-EIS (**b**) Proposed f-EIS.

Figure 2 depicts a scheme for detecting the Aβ in the f-EIS-based IME sensor: the antibody that specifically binds to the Aβ is locally immobilized on the surface of the insulator between the electrodes. The Aβ and the redox reagent, [Fe(CN)_6_]^3−/4−^, are included in the buffer.

In the reaction condition described as f-EIS in Figure 2a, the redox reagents simultaneously cause the charge transfer and the aggregation of Aβ, the antibody not only reacts with the aggregated Aβ but also with the unaggregated Aβ. This causes a biased change of *R*_ct_ of the f-EIS-based IME sensor, which consequently results in reduced sensitivity. To improve the sensitivity, we proposed a new detection method that preemptively controls the reaction environment with two types of buffer: one containing only the redox reagent for the measurement of the impedance change before and after the immunoassay and another composed of Aβ in phosphate buffered saline (PBS) buffer used during the Aβ immunoassay. The proposed method alleviates Aβ aggregation because the Aβ does not directly contact the redox reagent that causes the aggregation. Therefore, only the unaggregated Aβ binds with the antibody, as shown in Figure 2b, and the proposed f-EIS-based IME sensor can precisely detect the Aβ with high sensitivity.

## 4. Methods

### 4.1. Chemical

A purified anti-beta-amyloid, 1–16 antibody, 6E10 (Biolegend, San Diego, California, US), and amyloid beta (Aβ) (Sigma-Aldrich, Yongin-si, Gyenggi-do, Korea) were used for the immunoassay. The 6E10 was immobilized by using 1% (3-amino-propyl)triethoxysilane (APTES) (Sigma-Aldrich, Yongin-si, Gyeonggi-do, Korea), in an isopropanol alcohol (IPA) (Daejung Chemicals & Metals, Siheung-si, Gyeonggi-do, Korea), 2 mM poly(*N*-vinylpyrrolidone) with an aldehyde end-group (PVP-CHO) in sodium bicarbonate (NaHCO_3_) (Sigma-Aldrich), 10 mM sodium borohydride (NaBH_4_) (Sigma-Aldrich) in NaHCO_3_, and 1% glutaraldehyde (Daejung Chemicals & Metals, Siheung-si, Gyeonggi-do, Korea) in NaHCO_3_. The buffer solution for faradaic spectroscopy was composed of 25 mM redox reagent, [Fe(CN)_6_]^3−/4−^, in 10 mM PBS, which was a mixture of potassium ferricyanide (K_3_[Fe(CN)_6_]) (Sigma-Aldrich) and potassium ferrocyanide (K_4_[Fe(CN)_6_]·3H_2_O) (Sigma-Aldrich) with the same molarity. The buffer solution for nf-EIS was the 10 mM PBS itself with no dissolve solutes.

### 4.2. Interdigitated Microelectrode Sensor

**Figure 3:**
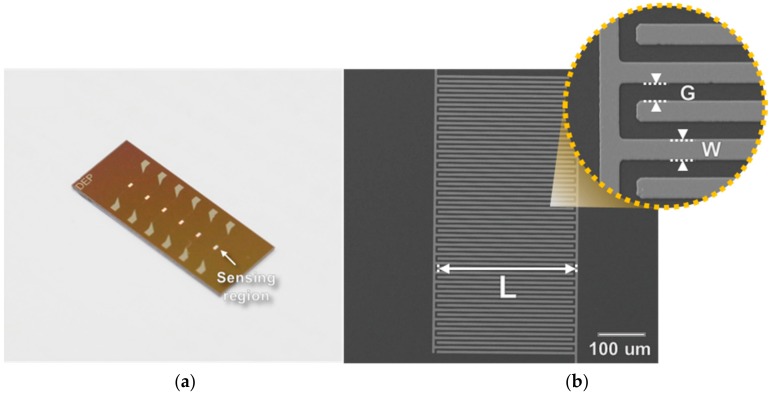
(**a**) Interdigitated microelectrode (IME) sensor for the detection of Aβ. (**b**) ×200 Scanning Electron Microscopy (SEM) image of the sensing region.

The sensor having six arrays of interdigitated microelectrodes (IMEs) was fabricated by the MEMS process (Figure 3a) [16]. First, an SiO_2_ layer with a thickness of 3000 Å was deposited via a thermal oxidation process for insulation, and a Ta/Pt with a thickness of 300/1500 Å was then deposited on SiO_2_ layer as the electrode layer via sputtering. Each IME sensor was composed of one sensing region with 30 pairs of micro-electrode arrays and two contact pads. A width (*W*) and a length (*L*) of one finger in the IME were 5 μm and 300 μm, respectively, and the gap (*G*) between each finger was 5 μm (Figure 3b).

For the detection of Aβ, the 6E10 was immobilized on the surface of the IME sensor (the SiO_2_ layer) by subsequent chemical treatments. The sensor was washed with a piranha solution (H_2_SO_4_:H_2_O_2_ = 4:1), deionized water (D.W.), and IPA to remove the organic and inorganic residues and functionalized by 1% (3-amino-propyl)dimetylethoxysilane (APDMES), PVP-CHO, 10 mM NaBH_4_, and 1% glutaraldehyde [16]. After antibody immobilization on the IME, a polydimethylsiloxane (PDMS) mold with a microfluidic channel was bonded onto the sensor.

### 4.3. Impedance Measurement

**Figure 4:**
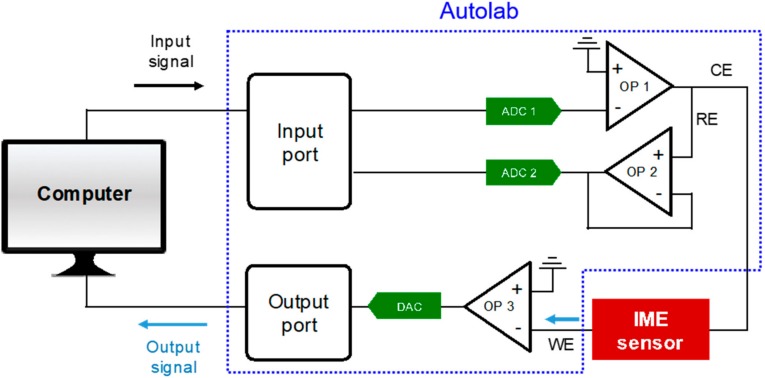
Impedance measurement set-up.

Figure 4 shows the measurement system to analyze the impedance spectrum of the IME sensor. The impedance analyzer, Autolab PGSTAT302N (Metrohm Autolab, Utrecht, Netherlands) consists of three output probes for impedance analysis: a counter electrode (CE), a reference electrode (RE), and a working electrode (WE), each probe transmits a signal through an operational amplifier (OP) and an analog-to-digital converter (ADC) or digital-to-analog converter (DAC). A working electrode (WE) and a counter electrode (CE) of the impedance analyzer were each connected to one of the electrode pads of the IME sensor and the impedance spectrum of the IME sensor was analyzed with electrochemistry software, NOVA (Version 2.1, Metrohm Autolab, Utrecht, Netherlands). A sinusoidal wave of 0.05 V with zero dc bias was applied to the IME sensor as an input signal, and the output signal, an impedance spectrum of the IME sensor, was measured at the frequency range from 10 Hz to 100 kHz.

### 4.4. Experimental Process

**Figure 5:**
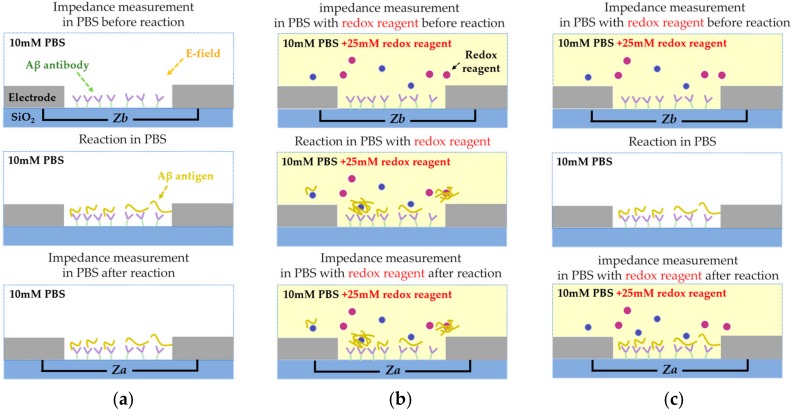
Illustration of experimental processes for (**a**) non-faradaic EIS (nf-EIS), (**b**) f-EIS, and (**c**) the proposed f-EIS.

The experiments were conducted with 3 different EIS-based IME sensors: (a) nf-EIS, (b) f-EIS, and (c) the proposed f-EIS. The experimental process of each sensor is illustrated in Figure 5. The first step involved measurement of impedance before the immunoassay (*Z*_b_). For the measurement of *Z*_b_, respective buffer solutions were injected into the PDMS fluidic channels: 10 mM PBS for nf-EIS; 10 mM PBS with 25 mM redox reagent for the conventional and proposed f-EIS. The next step was the addition of Aβ to initiate the immunoassay. The buffer solution containing Aβ was injected into the PDMS channels. In the case of the nf-EIS and f-EIS sensors, the buffer solutions in the immunoassay process were the same as the solution used in the first step, i.e., the measurement of *Z*_b_. In the proposed f-EIS, the buffer solution was changed from 10 mM PBS with 25 mM redox reagent to 10 mM PBS containing Aβ after the sensing region was rinsed to remove redox reagent residues completely. The immunoassay of Aβ lasted for 20 min at room temperature. After that, sensors were washed with their respective buffer solutions used in the immunoassay to remove Aβ residues that may have caused non-specific bindings. The last procedure was impedance measurement after the immunoassay (*Z*_a_) to obtain appropriate impedance changes. The impedance measurement was conducted in the same solution environment as the first step: 10 mM PBS for nf-EIS and 10 mM PBS with 25 mM redox reagent for f-EIS and the proposed f-EIS. The change in the impedance spectrum was analyzed by computing the difference Δ*Z* between *Z*_a_ and *Z*_b_.

## 5. Results and Discussion

### 5.1. The Non-Faradaic and Faradaic Electrochemical Impedance Spectroscopy-Based IME Sensors

**Figure 6:**
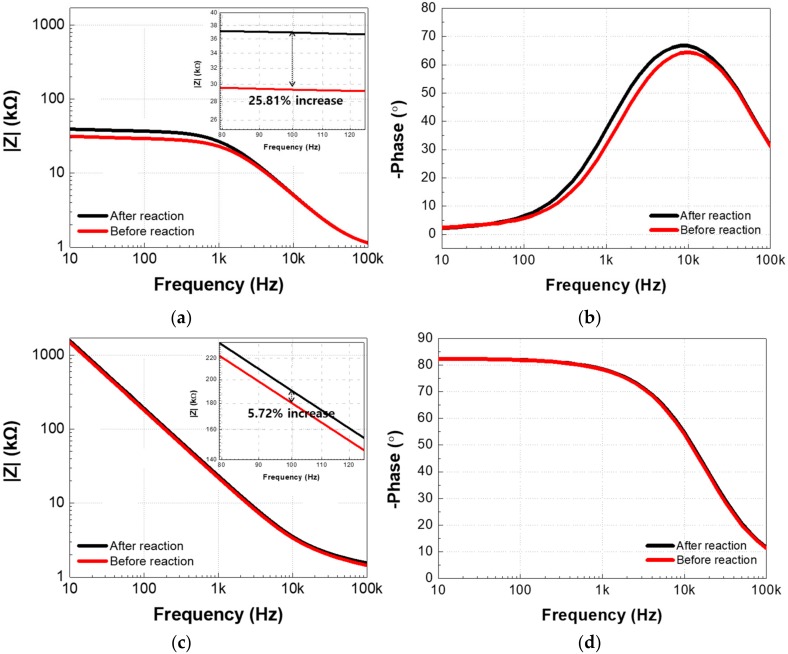

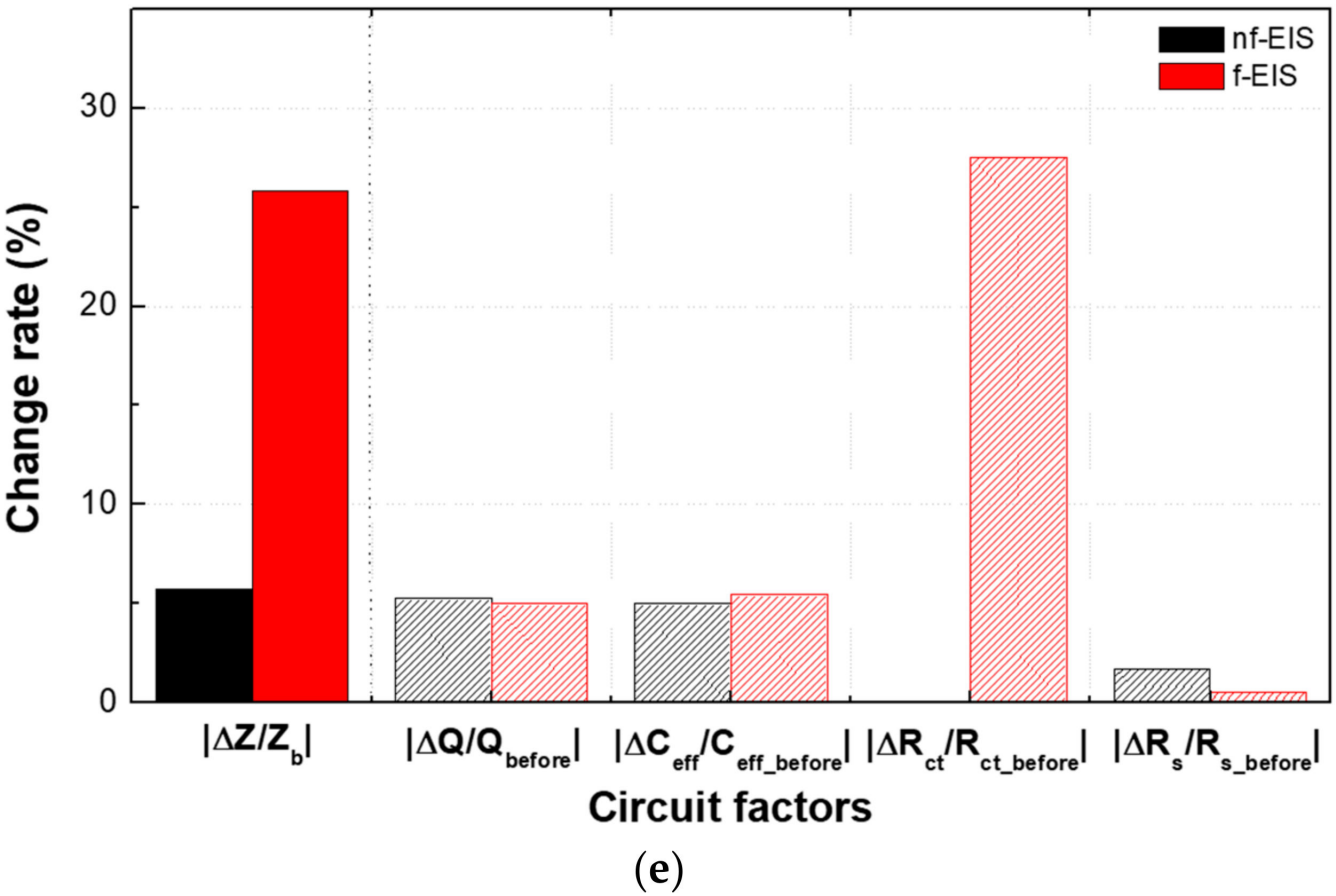
(**a**,**b**) Measured impedance spectrum (10 Hz–100 kHz) of nf-EIS and (**c**,**d**) f-EIS. (**e**) Percentage change of impedance magnitude at 100 Hz and equivalent circuit factors of the two EIS-sensors (The equivalent circuit fitting residual, r^2^ = 0.9802 for nf-EIS and r^2^ = 0.9137 for f-EIS).

Impedance spectra measured before and after Aβ antigen–antibody binding in the nf-EIS and f-EIS sensors were fitted to the equivalent circuits (Figure 2b,d, respectively). The fitting method was least mean square approximation. Figure 6a,b show the impedance spectrum bode plot of the nf-EIS sensor before and after the immunoassay with the antigen concentration of 100 pg/mL (the highest concentration used in the experiments). The impedance magnitude at 100 Hz, where capacitive impedance dominates, was increased by only 5.71% after the immunoassay, which is the reason why the curves (Figure 6c, |*Z*| vs. freq) appear to be overlapped on the log scale. For nf-EIS, the impedance increase at the low frequency can be estimated by the changes in the effective capacitance *C*_eff_, which is given by [17].

(2) $$C_{\mathrm{eff}}=Q^{\frac{1}{\alpha }}R_{s}{}^{\frac{\left(1-\alpha \right)}{\alpha }}\;\left(\mathrm{for}\;\mathrm{nf}-\mathrm{EIS}\right).$$

Equation (2) relates the parameter *Q* of the CPE, the solution resistance *R*_s_, and the scalar constant α. The interpolated value of *Q* was decreased from 15.2 ± 0.17 nFs^(α−1)^ to 14.4 ± 0.19 nFs^(α−1)^, and *R*_s_ was increased from 1580 ± 4.9 Ω to 1607 ± 3.6 Ω. The value of α remains at 0.915, which represents the surface uniformity. This results in the overall decrease in the effective capacitance from 5.66 ± 0.11 nF to 5.38 ± 0.12 nF (by 4.95%).

On the other hand, results of the f-EIS show a noticeable increase (by 25.8%) in the impedance magnitude (Figure 6c) for the same antigen concentration of 100 pg/mL. In faradaic spectroscopy, the impedance change primarily corresponds to the increase in the charge transfer resistance *R*_ct_ (28.7 ± 2.3 kΩ to 36.6 ± 2.7 kΩ). The other factors also changed after the immunoassay, excluding α = 0.93, *Q* from 22.2 ± 0.29 nFs^(α−1)^ to 21.1 ± 0.34 nFs^(α−1)^, and *R*_s_, from 920 ± 1.7 Ω to 925 ± 2.9 Ω. In the f-EIS, the *C*_eff_ can be calculated by Equation (3), and the change of *C*_eff_ changed from 9.76 ± 0.14 nF to 9.23 ± 0.18 nF (by 5.43%) [17].

(3) $$C_{\mathrm{eff}}=Q^{\frac{1}{\alpha }}{\left(\frac{R_{S}\cdot R_{\mathrm{CT}}}{R_{S}+R_{\mathrm{CT}}}\right)}^{\frac{1-\alpha }{\alpha }}\left(\mathrm{for}\;f-\mathrm{EIS}\right).$$

Figure 6c shows a bar chart comparing the two types of sensors regarding the change rate of the fitted equivalent circuit factors and impedance at 100 Hz. The changes in *C*_eff_ and *R*_ct_ dominated the overall impedance change for the nf-EIS and f-EIS, respectively. |∆*Q*/*Q*_before_| (or |∆*C*_eff_/*C*_eff_before_|) of the two sensors were of comparable values (~5%), while |∆*R*_s_/*R*_s_before_| was a minor factor (< 2%). In terms of the overall % impedance change, |Δ*Z*/*Z*_b_|, the f-EIS (25.81%) was 4.52-fold greater than nf-EIS (5.71%) due to the presence of *R*_ct_.

### 5.2. The Proposed Faradaic Electrochemical Impedance Spectroscopy -Based IME Sensor

Although the total impedance change was higher in the f-EIS sensor than the nf-EIS sensor, the f-EIS-based IME sensor without buffer control during the immunoassay was unsuited to detect the Aβ because of the aggregation of Aβ in the presence of Fe^2+^ in the buffer. The proposed f-EIS sensor, however, enabled the highly sensitive detection of Aβ without the Aβ aggregation. The aggregation of Aβ was alleviated by changing the buffer that contains the [Fe(CN)_6_]^3−/4−^ into the pure buffer without the [Fe(CN)_6_]^3−/4−^ during the reaction of Aβ with 6E10. The performance of the proposed f-EIS-based IME sensor was demonstrated by measuring the detection sensitivity of Aβ.

**Figure 7:**
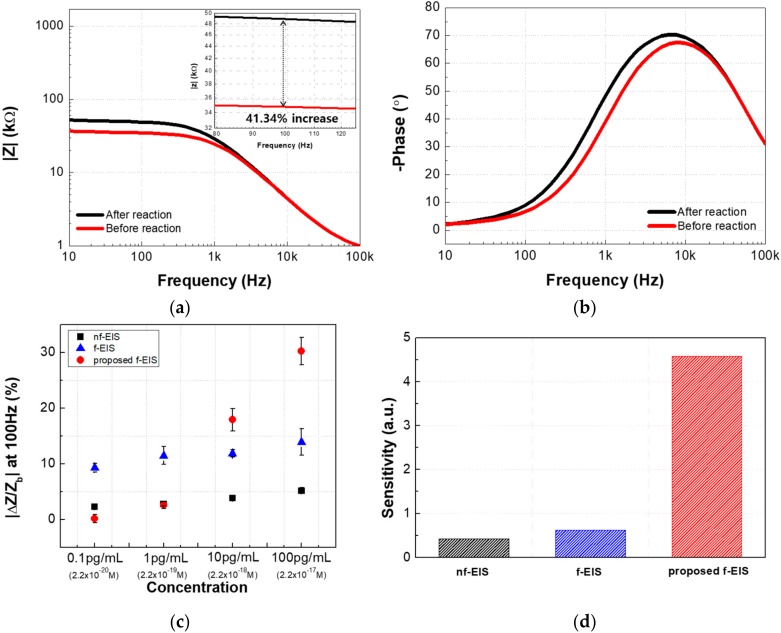
(**a**,**b**) Measured impedance spectrum (10 Hz–100 kHz) of the proposed f-EIS (the fitting residual r^2^ = 0.9248). (**c**) $\left|\Delta Z/Z_{b}\right|$ at 100Hz vs. concentration for the three methods. (**d**) Slope of Figure 7a ($\left|\Delta Z/Z_{b}\right|$ (*y*) vs. concentration (*x*) [pg/mL]); nf-EIS: *y* = 0.4231 × ln(*x*) + 3.0236, r^2^ = 0.9671; f-EIS: *y* = 0.6164 × ln(*x*) + 10.924, r^2^ = 0.9446; proposed f-EIS: *y* = 4.5859 × ln(*x*) + 7.4451, r^2^ = 0.9382.

Figure 7a,b show the bode plot of the proposed f-EIS before and after the immunoassay with an antigen concentration of 100 pg/mL. As shown in Figure 7a, the proposed f-EIS method can achieve a higher impedance change (41.3%) than the f-EIS method without buffer control (25.8%). In the proposed f-EIS-based IME sensor, the change of charge transfer resistance, |Δ*Rct*/*Rct*_before_|, was 43.48% (32.5 ± 1.6 kΩ to 52.5 ± to kΩ), about 1.5 times larger than that of the f-EIS (27.1%, 28.7 ± 2.3 kΩ to 36.6 ± 2.7 kΩ). The change rate of other factors of the proposed f-EIS were 4.1% for *Q* (21.7 ± 0.21 nFs^(α−1)^ to 20.8 ± 0.23 nFs^(α−1)^), 1.1% for *R*_s_ (853 ± 1.3 Ω to 862 ± 5.4 Ω), and 4.2% for *C*_eff_ (9.53 ± 0.11 nF to 9.13 ± 0.13 nF), excluding α = 0.93, which is the unchanged value.

The proposed f-EIS technique not only amplifies the impedance change at a specific concentration of Aβ but also enhances the detection sensitivity of the f-EIS sensor. Figure 7c indicates the impedance change according to the concentration of Aβ in the nf-EIS-based, the conventional f-EIS-based and the proposed f-EIS-based IME sensors, respectively. The impedance change, represented on the y-axis, was calculated by subtracting the background noise from the change in impedance by the reaction between the antibody and antigen at 100 Hz. Each value of noise was approximately 1.412 + 0.172 and 10.193 + 0.603 in the non-faradaic and faradaic case, respectively. The impedance changes were approximately 2.26 ± 0.28%, 2.79 ± 0.40%, 3.33 ± 0.42%, and 5.15 ± 0.54% for the concentration of Aβ ranged from 0.1 to 100 pg/mL, respectively, in the nf-EIS-based IME sensor. The changes in the impedance measured in the f-EIS-based IME sensor was measured to be approximately 9.31 ± 0.77%, 11.46 ± 1.59%, 11.85 ± 0.67%, and 13.91 ± 2.34% for the same sets of Aβ concentration, respectively. In the results, although the absolute impedance change rate of the f-EIS-based IME sensor was approximately 4-fold higher than the change of the nf-EIS-based IME sensor at all Aβ concentrations, there was no significant difference in the slope of the |Δ*Z*/*Z*_b_| according to concentration of Aβ between the nf-EIS and f-EIS sensors. On the other hand, in the proposed f-EIS-based IME sensor, the impedance changes drastically increased: 0.14 ± 0.72%, 2.61 ± 0.65%, 17.91 ± 2.01%, and 30.24 ± 2.43% according to the concentration of Aβ ranged from 0.1 to 100 pg/mL, respectively. Through the impedance change according to the concentration of Aβ, sensitivity in the three types of the EIS-based IME sensors was computed, respectively. The sensitivity referred to the slope of the linear relationship between |Δ*Z*/*Z*_b_| and the logarithmic value of Aβ concentration and was shown in Figure 7c. The sensitivities were approximately 0.42, 0.62, and 4.59 in the nf-EIS, the f-EIS, and the proposed f-EIS-based IME sensors, respectively. Thus, the sensitivity of the proposed f-EIS-based IME sensor was enhanced approximately 10.93-fold and 7.40-fold compared with the sensitivities of the nf-EIS-based and the f-EIS-based sensors, respectively. This shows that the proposed method enables the application of faradaic spectroscopy to the detection of Aβ with high sensitivity and minimizes the aggregation effect by deliberately controlling buffer solutions.

## 6. Conclusions

In this paper, we propose an f-EIS-based method that enhances the sensitivity of IME sensors for Aβ detection and minimizes aggregation. The proposed f-EIS method employs two key ideas: (1) detection using charge transfer resistance measurement in a buffer solution containing redox reagent, Fe(CN_6_)^3−/4−^; (2) deliberate control of the buffer environment during the immunoassay to alleviate aggregation. The buffer control process during the immunoassay prevents direct interaction between Aβ and the redox reagent, Fe(CN_6_)^3−/4−^, which is the primary cause of aggregation. The experimental results show that the proposed f-EIS method with buffer control enhanced Aβ detection sensitivity 10.93-fold and 7.40-fold, compared with the nf-EIS sensor and conventional f-EIS sensor, respectively. Therefore, the proposed f-EIS IME sensor enables sensitivity improvement by using charge transfer resistance and alleviating the aggregation effect. The detection sensitivity of the proposed f-EIS can be improved by modification of the sensing region in further studies; suggested methods include the immobilization of antibodies on electrode surfaces instead of on the insulator between electrodes. The antigen–antibody complex formation at the electrode surface can maximize the surface blocking effects of the complex, which would result in the amplified change of charge transfer resistance after the immunoassay.
